# Phase space electron hole in the Venusian upper mantle boundary

**DOI:** 10.1038/s41598-025-98769-4

**Published:** 2025-05-22

**Authors:** Ahmed Gamal, Waleed Moslem Moslem, Mohamed Saleh Yousef, Ali Yahia Ellithi

**Affiliations:** 1 Department of Physics,Faculty of Science, Cairo University, Giza, 12613 Egypt; 2 Department of Physics, Faculty of Science, Port Said University, Port Said, 42521 Egypt; 3 Centre for Theoretical Physics, The British University in Egypt (BUE), El-Shorouk, Cairo Egypt

**Keywords:** Ion-acoustic solitary waves, Double layer structure, Schamel–Korteweg–de Vries equation, Upper mantle boundary, Plasma physics, Planetary science, Space physics

## Abstract

The fluctuations based on the “phase space electron hole” in the Venusian upper mantle boundary due to the Parker Solar Probe (PSP) and Pioneer Venus Orbiter (PVO) observations are detected. This study examines the characteristics of ion-acoustic solitary waves and double-layer structures. The influence of various plasma parameters on the wave properties is investigated to gain a better understanding of the formation and propagation of these non-linear structures. The Schamel-KdV equation is derived using a hydrodynamic description, accounting for a small percentage of non-isothermal trapped electrons. A parametric investigation was then conducted, utilizing spacecraft observations of the plasma configuration at Venus, including observed data of densities, temperatures, and velocities. Solitary waves and double-layer structures can exist in two different modes: slow and fast. In order to identify which structure is most closely matched with PVO observation for Doppler-shifted frequencies of $\sim$ 5.4 kHz and an electric field of $\sim$ 15 mV/m, we examined these structures in the context of available data. It is found that our results of the ion-acoustic wave in fast mode are in agreement with the mission data from PSP and PVO.

## Introduction

Venus may be seen with both the naked eye and large telescopes from Earth. In terms of size and mass, it is Earth’s twin; Venus lacks an intrinsic planetary magnetic field, which results in the solar wind (SW) having an essential (direct) impact on the atmosphere of the planet. This leads to the formation of the ionosphere and creates an induced magnetosphere that is identical to Earth’s magnetosphere but insufficient to prevent solar wind energy and momentum from reaching Venus’ upper ionosphere directly in contrast to Earth. As a result, atmospheric erosion causes ionospheric ions to escape from the dayside, cross the terminator, and invade the nightside or tail^1,2^. This feature plays a crucial role in identifying the features of this planet’s plasma wave environment^3,4^. Ionospheres and magnetospheres are examples of space plasmas that generally constitute collisionless media. As a result, plasma waves transfer energy and momentum to other plasma areas. So they are regarded as the vital component of space plasmas. Waves have been discovered to be commonplace in space plasma because it is either always out of thermal equilibrium or contains a large amount of free energy. One basic mode of the plasma is the ion acoustic wave (IAW). The magnetosheath is one of the many locations in the planet’s environment where electrostatic solitary waves (ESWs) are seen^5^.

Many space missions have explored extensive research on the interaction between SW and Venus, including Venera 9 and 10, the Pioneer Venus Orbiter (PVO), and Venus Express (VEX). The last two are the most significant spacecraft that have frequently studied Venus’s ionosphere over many decades of years^6–8^. After VEX was safely launched into Venus’s orbit in the beginning of 2006, an enormous amount of data about the planet’s atmosphere from its surface to its uppermost layers was obtained. Venus is therefore the planet with the greatest aeronautical exploration^9^. During the PVO mission, the Orbiter Electric Field Detector (OEFD) measured plasma waves in the Venus mantle (between the magnetosheath and the lobe region). Different waves were seen during the PVO flybye, and it was determined that whistler waves were responsible for the wave observations in the 100 Hz channel and Doppler-shifted ion acoustic waves in the 730 Hz and 5.4 kHz channels^4,8,10^. Recent studies expand the depth and trustworthiness of investigations into wave phenomena in Venus’s environment. For example,^11^ presented a review on the plasma waves around Venus that cleared that around Venus, plasma waves influence particle scattering and energy loss while serving as diagnostic tools to determine plasma properties like electron density. However, only 4 out of 23 missions equipped with plasma wave detection instruments have thoroughly explored Venus. Observed waves include bow shock-related waves, but many predicted waves remain unobserved. Kamalam et al.^12^ studied the Venusian plasma environments of protons, oxygen ions, electrons, and solar wind particles. The ion acoustic waves (IAWs) kinetic dispersion was obtained. It is found that two wave modes are identified, the beam-driven mode, which depends on solar wind protons, and the ion-acoustic mode, which is driven by Venusian ions and modified by solar wind electrons. These results suggest that these modes could serve as potential candidates for interpreting electrostatic noise in the Venusian ionosphere at various frequencies. Rubia et al.^13^ presented a study on electrostatic solitary waves in the solar wind-driven Venusian ionosphere, utilizing a theoretical framework based on a multicomponent plasma comprising electrons and ions from both the solar wind and Venus. The research highlights key characteristics such as amplitude, width, and velocity, and predicts the presence of positive potential slow ion-acoustic solitons in two distinct altitude regions of Venus’ ionosphere. The findings are further correlated with observations from earlier missions, including the Pioneer Venus Orbiter and the Solar Orbiter. Morsi et al.^14^ investigated the propagation of nonlinear ion-acoustic fluctuations in Venus’s mantle, driven by discoveries of ion-acoustic solitary and double-layer formations made by the Pioneer Venus Orbiter and Parker Solar Probe. The nonlinear Zakharov-Kuznetsov equation is derived to study the dynamics of these waves under different plasma conditions using a hydrodynamic method and reductive perturbation theory, showing important structural features for both solitary waves and double-layers. In the frequency ranges relevant to earlier spacecraft observations, the results show the presence of low-frequency electrostatic activity, which are in line with the recent Parker Solar Probe measurements of the DLs propagating in the magnetosheath of Venus. George et al.^15^ presents findings from the Parker Solar Probe (PSP) during its Venus gravity assists (VGAs), which lower the probe’s perihelion while enabling high-resolution observations of plasma waves in Venus’s induced magnetosphere. PSP identified and mapped Langmuir, ion-acoustic, whistler-mode, and ion-cyclotron waves around Venus. Compared to earlier missions like the Pioneer Venus Orbiter (PVO) and Venus Express (VEX), the PSP’s FIELDS instruments offered unprecedented insights into wave properties, including power, bandwidth, and propagation direction-details previously beyond reach. These advancements illuminate wave generation mechanisms and Venus’s plasma environment, highlighting the critical role of modern plasma wave instrumentation in future missions.

Holes in space plasma are places where there are a lot fewer charged particles than in the surrounding plasma. Often called plasma voids or plasma cavities, holes in space plasma are locations where the density of charged particles is much lower than in the surrounding regions. Additionally, areas with a low to no magnetic field, known as “magnetic holes,” allow the redistribution of charged particles. One can classify these holes into three categories: (i) ion holes, which are locations in a plasma where there are many fewer positive ions (such as protons or heavier ions) than there are in the surrounding regions. A variety of the situations, such as turbulence within the plasma or interactions between the plasma and magnetic fields, can result in ion holes. (ii) Electron holes, which are regions where the electron density is lower than the surrounding plasma. Usually, strong electric fields or magnetic reconnection events that temporarily remove the electron population from a particular area can cause electron holes. Waves, instabilities, and particle trapping are examples of nonlinear interactions in plasma that are frequently linked to phase space holes. As a result of certain velocity distributions, they can occur in phenomena such as ion-acoustic holes as appeared in our observation. (iii) The phase space electron hole is a localized region of positive potential where the electron density is lower than the surrounding plasma. This happens because confined electron orbits reduce the phase-space density. The decreased electron density causes a local maximum in charge density, which in turn raises the electric potential. Consequently, the increased potential leads to a self-consistent increase in electron trapping.^16–20^.

The phase space density depletion of electrons by the bipolar electric field is the cause of phase space electron holes (EHs) which are electrostatic solitary waves in a bipolar parallel electric field^21^. Unlike ion holes, which have a negative potential, electron holes are localized positive potential structures, meaning that electrons are the trapped species. The hole solutions can be obtained in two distinct methods. Bernstein, Greene, and Kruskal refer to them as the “integral equation” and “differential equation” approaches. The integral approach is commonly referred to in the literature as the BGK approach, while the differential is frequently referred to as the Sagdeev, Schamel, or classical potential approach^16,22,23^. Phase space hole is a kinetic phenomenon, but there is a completely new perspective on understanding electron and ion holes seen in the phase space of the plasma that is offered by the fluid description of the phase space of the plasma particles^24,25^.

Ion-acoustic localized structures in the Venus nighttime or daytime ionosphere have consequences for planetary atmosphere research. Investigating these structures provides important historical context regarding the basic features of plasma physics. By using this information, we can gain a better understanding of the plasma processes occurring in Venus’s ionosphere. Examining ion-acoustic localized structures helps scientists understand the complicated interactions and processes occurring in Venus’s ionosphere. Recent observations in the induced magnetosphere of Venus have detected 30 kHz Langmuir oscillations, 5.4 kHz Doppler-shifted electrostatic ion-acoustic waves, and electromagnetic 100 Hz whistler waves^4,10,26^. One of these waves is mentioned as a phase space electron hole propagating parallel to the magnetic field with an electric field $\sim$ 15mV/m, and a Doppler-shifted frequency $\sim$ 5.4 kHz^13,21,27,28^.

This investigation aims to study the effect of phase space electron hole on the nonlinear evolution of IAWs at the upper mantle boundary. Based on the observed values of the temperatures and ion number densities recorded by VEX^29,30^, we construct a hydrodynamic fluid model that consists of two ionospheric positive ionic species (oxygen $O^+$ and hydrogen $H^+$) and a suitable electron distribution describing the phase space electron hole. The layout of this paper is organized as follows: In “Fluid model ”, it illustrates our model’s hydrodynamic equations. In “The evolution equation for small percentage non-isothermal electrons” , we will get the evolution equation for a small percentage of non-isothermal electrons. While in “Numerical analysis and discussion”, our theoretical study’s numerical results are explained. Finally, it is devoted to the summary and conclusion.

## Fluid model

According to space observations, we consider a collisionless, homogeneous, multispecies fluid plasma model comprising of two types of ionospheric positive ionic species (oxygen $O^+$ and hydrogen $H^+$) and inertia-less non-isothermal, ionospheric free, and trapped electrons.

The fluid equations (continuity and momentum equations) for oxygen ions

1a $$\begin{aligned} \frac{\partial n_o}{\partial t}+\frac{\partial n_o u_o}{\partial x}=0, \end{aligned}$$

1b $$\begin{aligned} \frac{\partial u_o}{\partial t}+u_o \frac{\partial u_o}{\partial x}+\frac{\partial \varphi }{\partial x}+3 \sigma _o n_o \frac{\partial n_o}{\partial x}=0, \end{aligned}$$

where $\sigma _o=\frac{T_o}{T_{e t}}$, is a temperature ratio between the oxygen ion $T_o$ and trapped electron $T_{e t}$.

The fluid equations for hydrogen ion

2a $$\begin{aligned} \frac{\partial n_h}{\partial t}+\frac{\partial n_h u_h}{\partial x}=0, \end{aligned}$$

2b $$\begin{aligned} \frac{\partial u_h}{\partial t}+u_h \frac{\partial u_h}{\partial x}+\rho _h \frac{\partial \varphi }{\partial x}+3 \sigma _h \rho _h n_h \frac{\partial n_h}{\partial x}=0, \end{aligned}$$

where $\sigma _h=\frac{T_h}{T_{e t}}$, is a temperature ratio between the hydrogen ion $T_h$ and trapped electron $T_{e t}$, $\rho _h=\frac{m_o}{m_h}$ is the mass ratio between the oxygen ion $m_o$ and the hydrogen ion $m_h$. The ionospheric electrons are assumed to be inertia-less and described by a small percentage of non-isothermal trapped electrons^31,32^.

3a $$\begin{aligned} n_{e}= & \exp (\varphi )-\frac{4}{3} b\varphi ^{\frac{3}{2}}, \end{aligned}$$

3b $$\begin{aligned} b= & \frac{1}{\sqrt{\pi }}\left( 1-\beta \right), \nonumber \\ \quad \quad  \nonumber \\ \beta= & \frac{T_{ef}}{T_{et}}\,, \end{aligned}$$

where *b* is a trapped electron coefficient and $\beta$ is a temperature ratio between the free and trapped electrons.

The Schamel model properly accounts for non-Maxwellian electron distributions like trapped electrons, explaining ion-acoustic wave dynamics in this plasma environment. This model is ideal for studying ion-acoustic waves when the electron distribution deviates from the Maxwell-Boltzmann profile due to trapping effects. The Schamel model’s main benefit is its ability to characterize plasma waves with phase space trapped electrons, which is the case at hand. Schamel’s model incorporates wave-potential well-trapped electrons. Trapped electrons alter wave dynamics and stability. In such cases, ion-acoustic waves have a nonlinear profile; Schamel fits better since it represents the nonlinearities.

The Poisson equation closes the set of Eqs. (1a)–(3b) as

4 $$\begin{aligned} \frac{\partial ^2\varphi }{\partial x^2}=\mu n_{e}-\alpha n_h-n_o, \end{aligned}$$

where $\mu =\frac{n_{e}^{(0)}}{n_o^{(0)}}$ and $\alpha =\frac{n_h^{(0)}}{n_o^{(0)}}$ are relative unperturbed densities. At equilibrium, the neutrality condition is given by

5 $$\begin{aligned} \mu =1+\alpha . \end{aligned}$$

The physical quantities in Eqs. (1a)–(4), $n_j$, $u_z$, $\varphi$, *x*, *t* are normalized by the unperturbed density $n_j^{(0)}$ of the jth species, ion acoustic speed $c_{so}=\left( \frac{k_BT_{et}}{m_o}\right) ^{\frac{1}{2}}$, the electrostatic potential $\frac{k_BT_{et}}{e}$, ion Debye length $$\lambda _D=\left( \frac{\epsilon _0\ k_BT_{et}}{\ n_o^{(0)}\ e^2}\right) ^{-\frac{1}{2}}\ $$, the inverse of ion plasma frequency ${w_{po}}^{-1}=\left( \frac{n_o^{(0)}\ e^2}{\epsilon _0\ m_o}\right) ^{-\frac{1}{2}}$, where *j* refers to plasma species ($H^+$, $O^+$, *e*), *z* refers to plasma species ($H^+$, $O^+$), $k_B$ is Boltzmann constant, and *e* is charge of electron.

## The evolution equation for small percentage non-isothermal electrons

To study the essential features of small- but finite amplitude IAWs, we use the reductive perturbation^33^. The extended space-time coordinate is presented by $\xi =\varepsilon ^\frac{1}{2} \left( x-\lambda t\right)$ and $\tau =\varepsilon ^\frac{3}{2} t$, where $\lambda$ is the linear phase velocity normalized by $c_{so}$, and $\varepsilon$ is a small parameter characterizing the smallness of the amplitude or dispersion of the nonlinear modes. The normalized physical quantities $n_j$, $u_z$, and $\varphi$ can be expanded around their equilibrium values in power series of $\varepsilon$ as

6 $$\begin{aligned} n_j=1+\varepsilon n_j^{\left( 1\right) }+\varepsilon ^2 n_j^{\left( 2\right) }+\varepsilon ^3 n_j^{\left( 3\right) }+\ldots , \end{aligned}$$

7 $$\begin{aligned} u_z=\varepsilon u_z^{\left( 1\right) }+\varepsilon ^2 u_z^{\left( 2\right) }+\varepsilon ^3 u_z^{\left( 3\right) }+\ldots , \end{aligned}$$

8 $$\begin{aligned} \varphi =\varepsilon \varphi ^{(1)}+\varepsilon ^2\varphi ^{(2)}+\varepsilon ^3\varphi ^{(3)}+\ldots . \end{aligned}$$

The reductive perturbation technique is essential for advancing both theoretical research and practical applications because it reduces the complexity of nonlinear systems to fundamental universal characteristics, especially in fields like fluid mechanics, plasma physics, and nonlinear optics. This method has important features:

(i) Simplify complex, high-dimensional nonlinear systems into manageable evolution equations, such as the Korteweg-de Vries (KdV) or Burgers equations. These simplified equations make it simpler to study the underlying physics by capturing the most significant elements of slow-time and long-wavelength phenomena,
(ii) It provides qualitative models for long-scale dynamics.
(iii) It is suitable for studying coherent structures, including solitons, shock waves, and wave envelopes-which are essential to many natural and artificial systems-can be studied, additionally, it offers a framework for investigating how non-linearity and dispersion interact, providing information on energy transfer, stability, and wave propagation.

It is important to remember that the perturbation of an equilibrium state in the plasma species is what leads to the production of IAWs. These disturbances are mostly caused by number densities, fluid velocities, and electrostatic potential. Therefore, the description of the perturbed fluid of the plasma species is already considered when developing the current model. We take a small percentage of trapped electrons, $b=\acute{b}\varepsilon ^\frac{1}{2}$ then apply the stretching space-time coordinates and expansion Eqs. (6)–(8) into fluid Eqs. (1a)–(4). The lowest-order equations $\varepsilon$ are

9 $$\begin{aligned} u_h^{\left( 1\right) }=\frac{\lambda \rho _h}{\lambda ^2-3\sigma _h\rho _h}\varphi ^{(1)}\,,\ \ \ \ \ n_h^{\left( 1\right) }=\frac{\rho _h}{\lambda ^2-3\sigma _h\rho _h}\varphi ^{(1)}, \end{aligned}$$

10 $$\begin{aligned} u_o^{\left( 1\right) }=\frac{\lambda \ }{\lambda ^2-3\sigma _o}\ \varphi ^{(1)}\,,\ \ \ \ \ \ \ n_o^{\left( 1\right) }=\frac{1}{\lambda ^2-3\sigma _o}\ \varphi ^{(1)}, \end{aligned}$$

11 $$\begin{aligned} n_{e}^{\left( 1\right) }=\varphi ^{(1)}. \end{aligned}$$

The compatibility condition is given by the Poisson equation as

12 $$\begin{aligned} \mu -\frac{\alpha \rho _h}{\lambda ^2-3\sigma _h\rho _h}-\frac{1}{\lambda ^2-3\sigma _o}=0. \end{aligned}$$

By considering the next-higher order terms $\varepsilon$ in the perturbation expansion, a system of equations is obtained that describes the dynamics of the second-order perturbed quantities. This system of equations can be solved using Eqs. (9)–(11) to derive the Schamel and Korteweg-de Vries (Schamel-KdV) equation,

Ion-acoustic waves in plasma are described mathematically by the Schamel-KdV equation, especially in cases where electron trapping is important. In this context, non-isothermality refers to deviations from the isothermal condition, which is the assumption that the electron temperature remains constant. A small percentage of non-isothermality is added to explain slight variations in thermal characteristics resulting from the existence of both trapped and free (Maxwellian) electron populations. The coefficient representing the trapped electrons is $\acute{C}$, while the coefficient representing the Maxwellian or non-trapped electrons is $\acute{A}$. Thus, the Schamel-KdV equation contains both of $\acute{A}$ and $\acute{C}$

13 $$\begin{aligned} \frac{\partial \varphi }{\partial \tau }+\left[ \acute{C}\sqrt{\varphi }+\acute{A}\ \varphi \right] \frac{\partial \varphi }{\partial \xi }\ +D\frac{\partial ^3\varphi }{\partial \xi ^3}=0\,, \end{aligned}$$

where $\acute{C}=CD, \ \acute{A}=DA$, *A* and *C* are the coefficients of the nonlinearity, *D* is the coefficient of the dispersion and given by

14 $$\begin{aligned} D=\frac{1}{\left[ \frac{2\alpha \lambda \rho _h}{\left( \lambda ^2-3\sigma _h\rho _h\right) ^2}+\frac{2\lambda }{\left( \lambda ^2-3\sigma _o\right) ^2}\right] },\ \ C=2b\mu ,\ \ A=\frac{3\lambda ^2+3\sigma _o}{\left( \lambda ^2-3\sigma _o\right) ^3}+\frac{3\lambda ^2\alpha {\rho _h}^2+3\alpha \sigma _h{\rho _h}^3}{\left( \lambda ^2-3\sigma _h\rho _h\right) ^3}-\mu . \end{aligned}$$

By using the space-time transformation, $\eta =\xi -M\tau$, the localized solution of Eq. (13) is given by^34–36^

15 $$\begin{aligned} \varphi _s=\left[ \frac{4CD}{15M}+\sqrt{\frac{16C^2D^2}{225M^2}+\frac{AD}{3M}}\cosh {\left( \sqrt{\frac{M}{4D}}\ \eta \right) }\ \right] ^{-2}. \end{aligned}$$

Special cases arise when $\acute{C}=0$ or $\acute{A}=0$, both representing distinct physical conditions influenced by electron trapping. When no trapped electrons are in the plasma, $\acute{C}=0$, the Maxwellian electron population completely regulates its behavior. Under these circumstances, the equation simplifies to a normal KdV form, producing nonlinear wave structures like solitons, which are impacted merely by the Maxwellian electron distribution without the special effects provided by electron trapping. This problem is relevant in plasmas in cases where the potential wells are too weak to effectively trap electrons or in cases when trapping mechanisms are negligible. On the other hand, Maxwellian electron impact is abolished, and the equation reduces to define a plasma dominated by fully trapped electrons when $\acute{A}=0$. Maximizing the effects of electron trapping in this highly nonlinear zone leads to the production of unusual wave structures, such as double layers. This condition emerges in plasmas with strong potential wells that confine a significant proportion of the electron count, highlighting the non-Maxwellian nature of their behavior. In the case of fully trapped electrons, i.e., $\acute{A}=0$, Eq. (13) becomes Eq. (16) with the solution^32,34^

16 $$\begin{aligned} & \frac{\partial \varphi }{\partial \tau }+\acute{C}\sqrt{\varphi }\frac{\partial \varphi }{\partial \xi }\ +D\frac{\partial ^3\varphi }{\partial \xi ^3}=0,  \nonumber \\ & \varphi _{Sch}=\frac{225\ M^2}{64C^2D^2}\textrm{sech}^4{\left( \sqrt{\frac{M}{16D}}\ \eta \right) }. \end{aligned}$$

For Maxwellian electrons, when we set $\acute{C}=0$, Eq. (13) transforms into Eq. (17) with the corresponding solution

17 $$\begin{aligned} & \frac{\partial \varphi }{\partial \tau }+\acute{A}\ \varphi \frac{\partial \varphi }{\partial \xi }\ +D\frac{\partial ^3\varphi }{\partial \xi ^3}=0,  \nonumber \\ & \varphi _{KdV}=\frac{3M}{AD} \textrm{sech}^2{\left( \sqrt{\frac{M}{4D}}\ \eta \right) }. \end{aligned}$$

The electric field $E_s$ associated with solitary waves from Eq. (15) is given by

18 $$\begin{aligned} E_s=-\frac{\sqrt{\frac{M}{D}} \sqrt{\frac{A D}{3 M}+\frac{16 C^2 D^2}{225 M^2}} \sinh \left( \frac{1}{2} \eta \sqrt{\frac{M}{D}}\right) }{\left[ \sqrt{\frac{A D}{3 M}+\frac{16 C^2 D^2}{225 M^2}} \cosh \left( \frac{1}{2} \eta \sqrt{\frac{M}{D}}\right) +\frac{4 C D}{15 M}\right] ^3}. \end{aligned}$$

The present work combines Schamel distributions to consider trapped electrons in a plasma system consisting of two positive ion species. Through analysis of nonlinear wave structures under non-Maxwellian electron populations, the model provides a more comprehensive and wide framework than that currently available. This improved model offers better insights into plasma dynamics, therefore enabling a more complete knowledge of nonlinear wave behavior in challenging plasma settings. Particularly in the context of ion-acoustic solitary waves, the complexity is much reduced by simplifying the model to a single ionic population with Schamel-distributed electrons, as investigated by^32^. This allows a targeted study of the interaction between a single ionic population and the influence of trapped electrons.El-Labany et al.^37^ studied cylindrical ion-acoustic solitons in positive-negative ions plasma with two-temperature trapped electrons. It constructs cylindrical Shamel-Korteweg-de Vries equation using reductive perturbation theory and investigates compressive and rarefactive solitons under diverse settings. Alternatively, the electron response is assumed to follow thermal equilibrium when the model is limited to two ion species with Maxwellian electrons as examined by^38^. This change simplifies the mathematical framework by excluding the effects of electron trapping, so stressing the importance of mass and density variations between the two ion species in wave dynamics and emphasizing the interactions among several ion modes in the absence of non-Maxwellian characteristics.

Within the framework of plasma physics, nonlinear waves, and other fields requiring complicated dynamical systems, the Schamel model has been thoroughly investigated. Often used to investigate events like electron-acoustic waves, soliton dynamics, and turbulence, the model offers a framework for characterizing nonlinear interactions in plasma waves. Scientists have investigated many kinds of solutions, each providing unique understanding of the nature of these nonlinear systems. Among the main solution forms in the Schamel model are solitons. The Schamel-KdV (SKdV) equation forecasts nonlinear occurrences in numerous physical contexts, therefore enabling several soliton solutions. The W-shaped soliton is one rather unique solution class^39^. The W-shaped soliton more dynamically depicts nonlinear wave interactions than single and shock wave solutions by virtue of its unique wave structure with many peaks and troughs. This soliton generated from Darboux shows in the SKdV framework the interactions between nonlinearity and dispersion. In space plasma settings, W-shaped solitons are very important as predicted nonlinear wave profiles. W-shaped solitons may help to describe intricate space plasma formations produced by nonlinear interactions between charged particles and waves. Their multi-peaked shape is most suited to describe the dispersion of nonlinear ion-acoustic waves produced in space plasma systems by electron entrapment and dispersion events. Nonlinear and dispersive coefficients allow one to modify W-shaped soliton solutions to reveal fresh dynamics and wave features. Including W-shaped solitons improves the applicability of the SKdV equation in plasma physics by extending the application of it to complex wave behavior and thereby enhances the spectrum of solutions. Moreover, this interesting class of solutions shows motivating future research. In our next study, we will examine the mathematical and physical effects of W-shaped solitons in space plasma settings. This implies looking at their stability, linkages, and involvement in multidimensional plasma systems with nonlinear and dispersive characteristics. These investigations will progress nonlinear wave study and enable us to comprehend the dynamics of plasma waves. A typical model for ion-acoustic waves in plasma, the Schamel-KdV equation has new precise and solitary wave solutions derived using the modified Kudryashov technique^40^. The findings emphasize the adaptability of the approach in handling nonlinear evolution issues in many physical environments. Emphasizing electron trapping in ion-acoustic wave interactions,Hussain A et al.^41^ examined the integrability characteristics of the Schamel-KdV equation. With more general relevance in mathematical physics, novel analytical solutions are constructed utilizing Lie symmetry analysis, the modified auxiliary equation process, and the extended Jacobi elliptic function expansion method. Investigating a new two-mode form of the coupled Schamel-KdV equations, Alquran^42^ modeled associated wave propagation in nonlinear optics and plasma physics. The work improves knowledge of wave propagation by offering new perspectives on wave dynamics affected by nonlinearity, dispersion, and phase velocity.

Double layers (DLs) are localized structures made up of two parallel, oppositely charged sheets that develop in a plasma. These DLs show a significant potential difference between the two sheets, which leads to a concentrated, rather strong electric field inside the DL zone. A particle’s interaction with the DL potential structure might cause it to accelerate, decelerate, or drift, depending on its starting direction of motion. Controlling charged particle mobility is a crucial characteristic of electric double layers in plasmas^43^.

By using the space-time transformation $\eta =\xi -M\tau$, the double-layer solution of Eq. (13) is given by^44^

19 $$\begin{aligned} \varphi _D=\ \frac{4{C}^2}{25{A}^2}\left[ 1-\tanh \left( {\eta }{\sqrt{\frac{M}{16D}}}\right) \right] ^2. \end{aligned}$$

The electric field $E_D$ due to the double-layer solution is

20 $$\begin{aligned} E_D=-\frac{2\sqrt{M}{C}^2}{25\sqrt{D}{A}^2}\left[ 1-\tanh \left( {\eta }{\sqrt{\frac{M}{16D}}}\right) \right] \text {sech}^2\left( {\eta }{\sqrt{\frac{M}{16D}}}\right) . \end{aligned}$$

## Numerical analysis and discussion

Our goal is to compare the characteristics of ion-acoustic waves in the upper mantle boundary with the observed wave frequencies and DL structures with data from PVO and PSP missions. To make this analysis, we will utilize the observational data of plasma parameters provided by the VEX spacecraft. Typical plasma configurations in the transition region, which is the focus of our study include are $\alpha \in \left[ 0.1,0.9\right]$, $\sigma _h \in \ \left[ 0.03,0.2\right]$, $\sigma _o \in \ \left[ 0.1,0.2\right]$ and $\beta \ \in \ \left[ 0.01,0.9\right]$^29,30^.

**Fig. 1:**
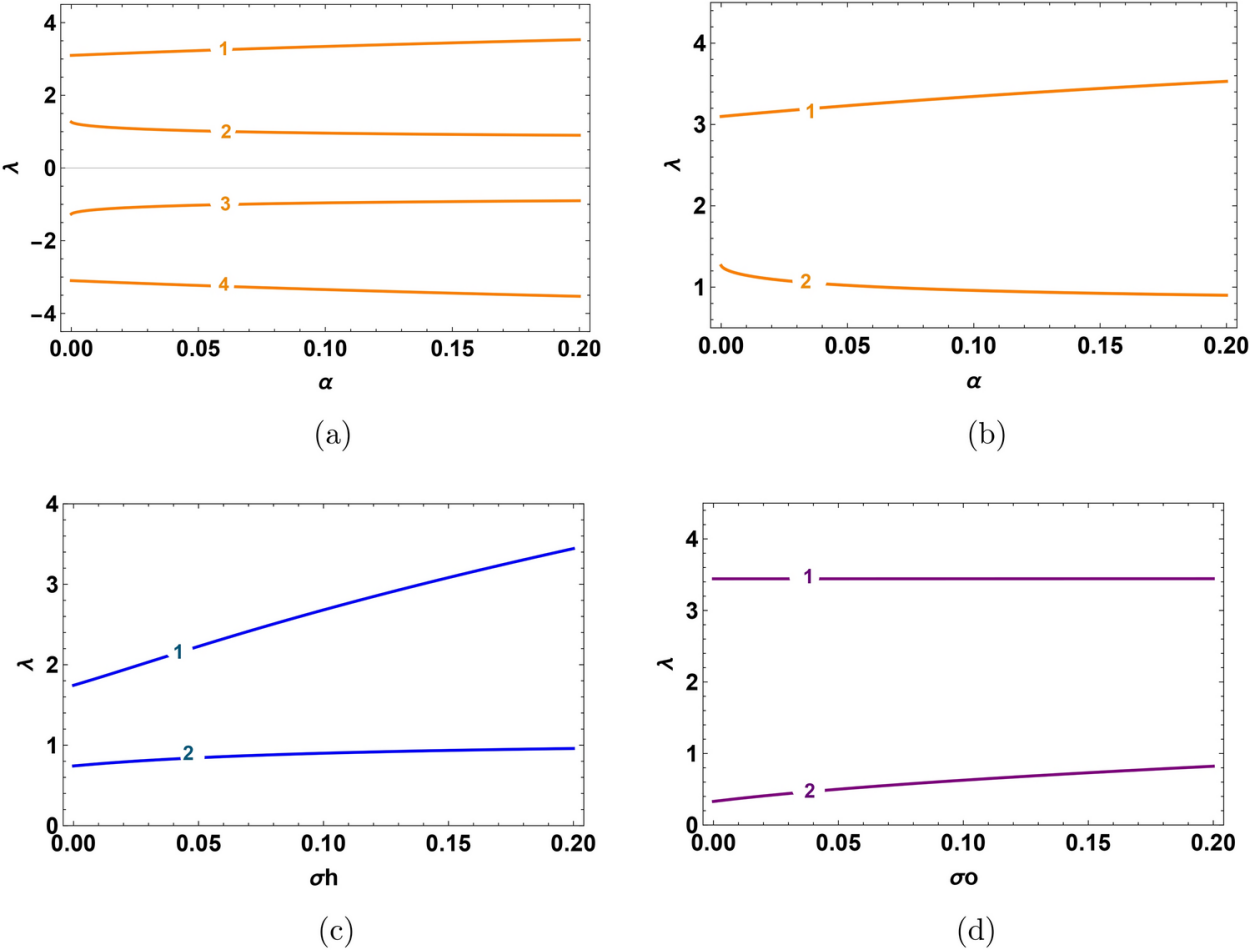
Variation of phase velocity with plasma parameters which $\lambda _1$, $\lambda _2$ in forward mode and $\lambda _3$, $\lambda _4$ in backward mode. (**a**) For the variation of density ratio $\alpha$ at $\sigma _h=0.2$, $\sigma _o=0.1$ and $\beta =0.55$ in fast mode, and $\beta =0.75$ in slow mode. (**b**) For the variation of density ratio $\alpha$ at $\sigma _h=0.2$, $\sigma _o=0.1$ and $\beta =0.55$ in fast mode and $\sigma _h=0.04$, $\sigma _o=0.2$ and $\beta =0.75$ in slow mode. (**c**) For the variation of temperature ratio $\sigma _h$ at $\alpha =0.15$, $\sigma _o=0.1$ and $\beta =0.55$ in fast mode and $\sigma _o=0.2$, $\alpha =0.8$ and $\beta =0.75$ in slow mode. (**d**) For the variation of temperature ratio $\sigma _o$ at $\alpha =0.15$, $\sigma _h=0.2$ and $\beta =0.55$ in fast mode and $\sigma _h=0.04$, $\alpha =0.8$ and $\beta =0.75$ in slow mode.

Based on the compatibility condition (12), we obtain four distinct roots, each of which denotes a possible phase velocity mode $\lambda$. In Fig. 1a, there are four modes: two forward modes $\lambda _1$, $\lambda _2$ and two backward modes $\lambda _3$, $\lambda _4$. Forward modes are classified into fast ion-acoustic mode $\lambda _1$ (corresponding to $H^+$ species) and slow ion-acoustic mode $\lambda _2$ (corresponding to $O^+$ species). The plasma parameters $\alpha$, $\sigma _o$ and $\sigma _h$ affected on the four modes. Lighter and quicker species are associated with faster modes, while heavier and slower species are associated with slower modes. When the density ratio $\alpha$ increases, the phase velocity $\lambda _1$ becomes slightly faster in fast mode while phase velocity $\lambda _2$ in slow mode decreases slightly, as shown in Fig. 1b. In Fig. 1c, when the hydrogen temperature ratio $\sigma _h$ increases, the thermal energy of hydrogen ions increases. Thus, the fast mode phase velocity $\lambda _1$ becomes fast, and $\lambda _2$ in slow mode, it increases very low slightly. During increasing the oxygen temperature ratio $\sigma _o$, the thermal energy increases, and slow mode phase velocity $\lambda _2$ increases, while phase velocity $\lambda _1$ in fast mode is not significantly changed, as shown in Fig. 1d. The same behavior is observed for backward modes due to the effect of the plasma parameters.

There are two solutions for the Shamel-KdV equation (13), i.e., solitary and double-layer structures. We will study the characteristics of these structures and examine how the plasma parameters affect these profiles.

### Solitary wave

**Fig. 2:**
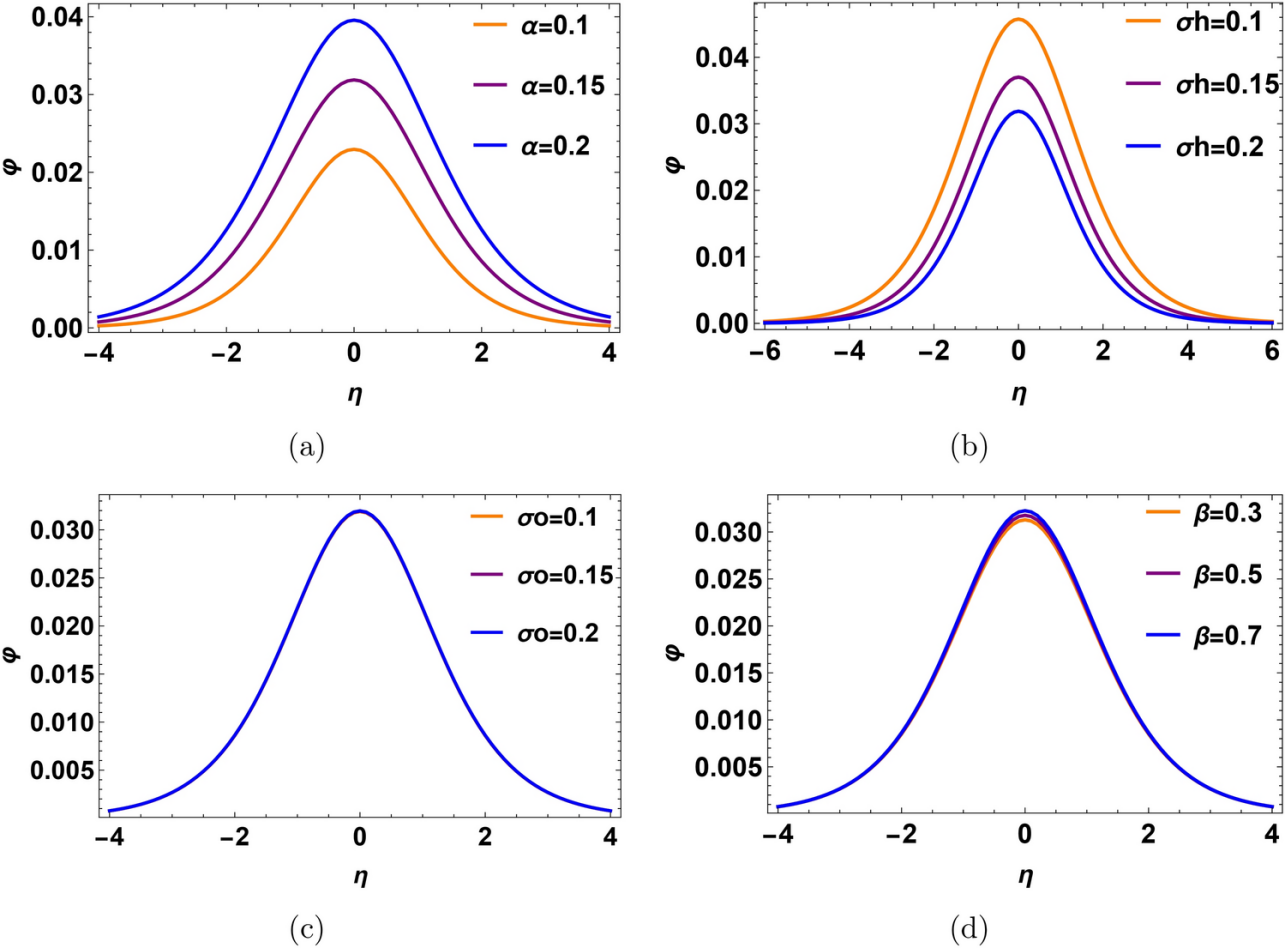
Profile of the electrostatic potential with different physical parameters in solitary waves, (**a**) the profile for the variation of density ratio $\alpha$ at $\sigma _h=0.2$, $\sigma _o=0.1$ and $\beta =0.55$. (**b**) The profile for the variation of temperature ratio $\sigma _h$ at $\alpha =0.15$, $\sigma _o=0.1$ and $\beta =0.55$. (**c**) The profile for the variation of temperature ratio $\sigma _o$ at $\alpha =0.15$, $\sigma _h=0.2$ and $\beta =0.55$. (**d**) The profile for the variation of temperature ratio of trapped coefficient $\beta$ at $\alpha =0.15$, $\sigma _o=0.1$ and $\sigma _h=0.2$.

In Fig. 2, the solitary pluse potential is investigated with different plasma parameters. Increasing the density ratio $\alpha$, make the pulse taller and wider as shown in Fig. 2a. The increase in density ratio $\alpha =\frac{n_h^{(0)}}{n_o^{(0)}}$ means that an abundance of hydrogen ions compared to oxygen ions. Leading to an increase in ion acoustic speed, implications for the energy transport and particle acceleration, this appears in high amplitude. On the contrary, as shown in Fig. 2b, increases in hydrogen temperature lead to a decrease in the amplitude of the solitary wave. Physically, the increase of $\sigma _h$ means that an access of hydrogen thermal temperature exists, i.e., hydrogen temperature increases. Our time scale is relative to oxygen ions since we make normalization with respect to ${w_{po}}^{-1}$ and $c_{so}$. Increasing the hydrogen temperature makes the hydrogen ions have high thermal energy then the thermal energy gap between oxygen and hydrogen increases, so the thermal energy enhances. Consequently, the possible interaction between hydrogen ions and oxygen ions becomes weak and the hydrogen ions can not interact effectively with oxygen ions. The solitary pulse in the oxygen ion time scale lacks energy due to increasing the relative thermal motion between hydrogen ions and oxygen ions. In Fig. 2c, d, the profile of pulses does not change with variation in the oxygen temperature ratio $\sigma _o$ and trapped electrons coefficient $\beta$. Since we use phase velocity $\lambda _1$ in fast mode, it is not dependent on $\beta$ and $\sigma _o$.

**Fig. 3:**
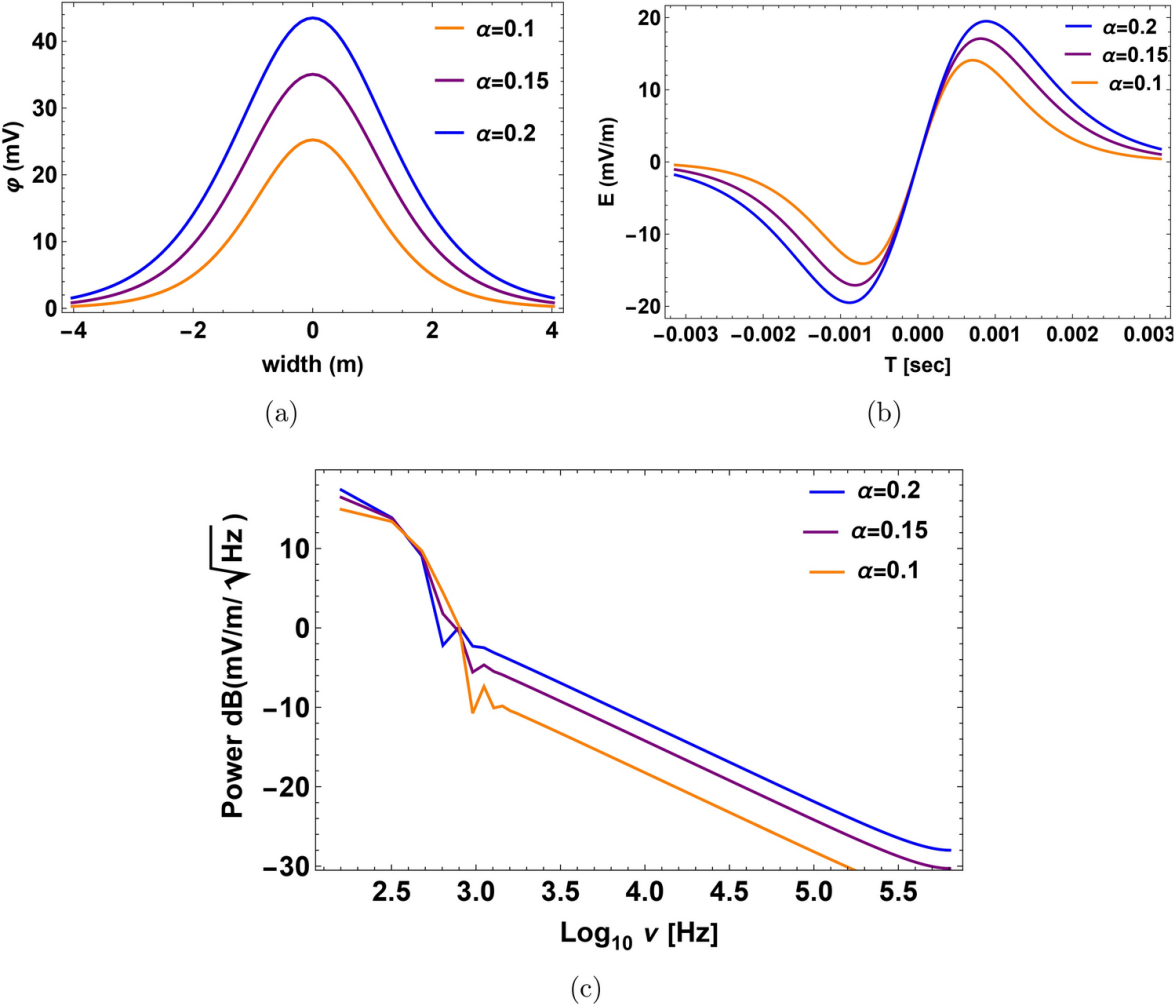
Profile of the solitary pulse with its corresponding bipolar electric pulse and the FFT power spectra at different relative densities $\alpha$ at $\sigma _h=0.2$, $\sigma _o=0.1$ and $\beta =0.55$. (**a**) Solitary pulse electrostatic potential expressed in millivolts (mV) with the width of the pulse in meters (m). (**b**) Associated electric field pulse with time duration in seconds (s). (**c**) The electric field power spectra obtained from the corresponding fast Fourier transform (FFT). The x-axis represents the frequency $log_{10}{\nu }$ in Hz, while the y-axis represents the strength of the electric field $dB(mV/m/\sqrt{Hz})$.

Figure 3a, b represent the non-normalized electrostatic potential with a spatial width and the electric field when relative densities change. When the density of hydrogen rises, the amplitude of the solitary wave, electric field, and time duration grow. By “duration,” we mean the pulse time duration of the nonlinear structure, which refers to the time it takes for the plasma structure to pass by the spacecraft’s instruments. If the calculated pulse time duration is greater than or equal to the observed time duration of the structures by the spacecraft’s instrument, then our model can foresee the observed structure. Otherwise, when the calculated pulse time duration is less than the time duration measured by the spacecraft’s instrument, it means that our model does not accurately predict the observed structure. The bipolar pulse’s fast Fourier transform (FFT) produces a broadband electrostatic activity with a frequency range of around (1–1.5) kHz as seen in Fig. 3c, we observe that decreasing frequency is correlated with rising relative density $\alpha$. Our results show that the electric field $E=(15$–20) mV/m, the time duration $T=(1$–1.6) ms and the frequency $f=(1$–1.5) kHz as shown in Fig. 3. These results could have implications for the electrostatic activity measurements made by the electric dipole aboard PVO in the Doppler-shifted frequency range of 5.4 kHz (i.e., the frequency $\sim$ 1.6 kHz without Doppler shifted with a bandwidth of the center frequency is ±15%), carried out by the electric dipole onboard PVO^13,21^. The mantle waves observed in the 5.4 kHz channels can be correctly interpreted as IAWs because our theoretical results show that IAWs should arise at frequencies where OEFD would have observed them. This is true even though no simultaneous electric and magnetic field observations were made with PVO and the frequency coverage of the electric field observations was noncontinuous. Solitary structures have recently been found using PSP on Venus in the upper mantle boundary^21,26,27^. The calculations of Rubia, the electric field $E=(0.03$—27.6) mV/m, time duration $T=(0.34$—22) ms and the frequency $f=9.8$ Hz — 8.7 kHz^13^. The calculations of morsi with the electric field $E=8$ mV/m, time duration $T=1.6$ ms and the frequency $f=6.3$ Hz —3.16 kHz^14^. Regarding ion acoustic wave in slow mode, the results are in disagreement with the observation.

### Double layer (DL) wave

Now, we shall investigate the DL, also known as the shock-like wave which is another type of nonlinear wave structure as seen in solution (19). Since this wave structure is known to produce powerful electric fields, it often acts as a way for particle acceleration. We will get the profile of DL pulses and their dependence on plasma parameters for two modes (i.e., fast and slow modes).

#### Fast mode

**Fig. 4:**
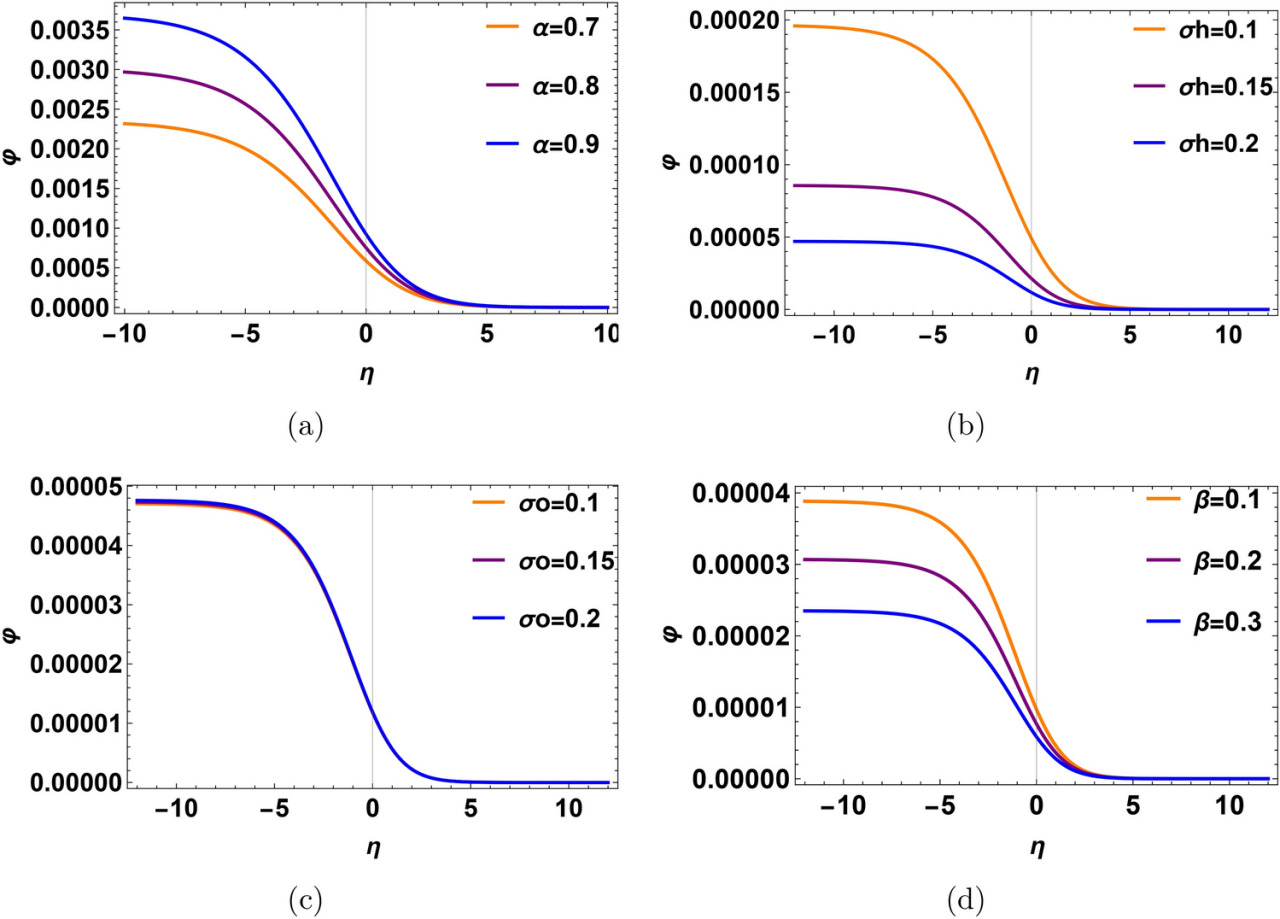
Profile of the electrostatic potential with different physical parameters in Double layer (DL). (**a**) The profile for the variation of density ratio $\alpha$ at $\sigma _h=0.2$, $\sigma _o=0.1$ and $\beta =0.01$. (**b**) The profile for the variation of temperature ratio $\sigma _h$ at $\alpha =0.15$, $\sigma _o=0.1$ and $\beta =0.01$. (**c**) The profile for the variation of temperature ratio $\sigma _o$ at $\alpha =0.15$, $\sigma _h=0.2$ and $\beta =0.01$. (**d**) The profile for the variation of temperature ratio of trapped coefficient $\beta$ at $\alpha =0.15$, $\sigma _o=0.1$ and $\sigma _h=0.2$.

In Fig. 4, we show the profile of the DL electric potential with different plasma parameters. One notices that as the relative density $\alpha$ increases, the profile of DL amplifies as shown in Fig. 4a. An increased density ratio $\alpha$ signifies more hydrogen ions relative to oxygen ions which raises ion acoustic speed. This affects energy transport and particle acceleration, resulting in higher amplitude. On the contrary, in Fig. 4b, when the hydrogen temperature ratio $\sigma _h$ rises, the DL profile becomes lower. As $\sigma _h$ increases, indicating a rise in hydrogen thermal temperature (hydrogen ions have high energy relative to oxygen ions according to phase velocities between them), the thermal energy gap between hydrogen and oxygen ions is higher. This reduces the effective interaction between hydrogen and oxygen ions due to the higher thermal energy of hydrogen ions, but our time-scale is relative to oxygen ions. So, the DL profile decreases (c.f. Fig. 2b).

**Fig. 5:**
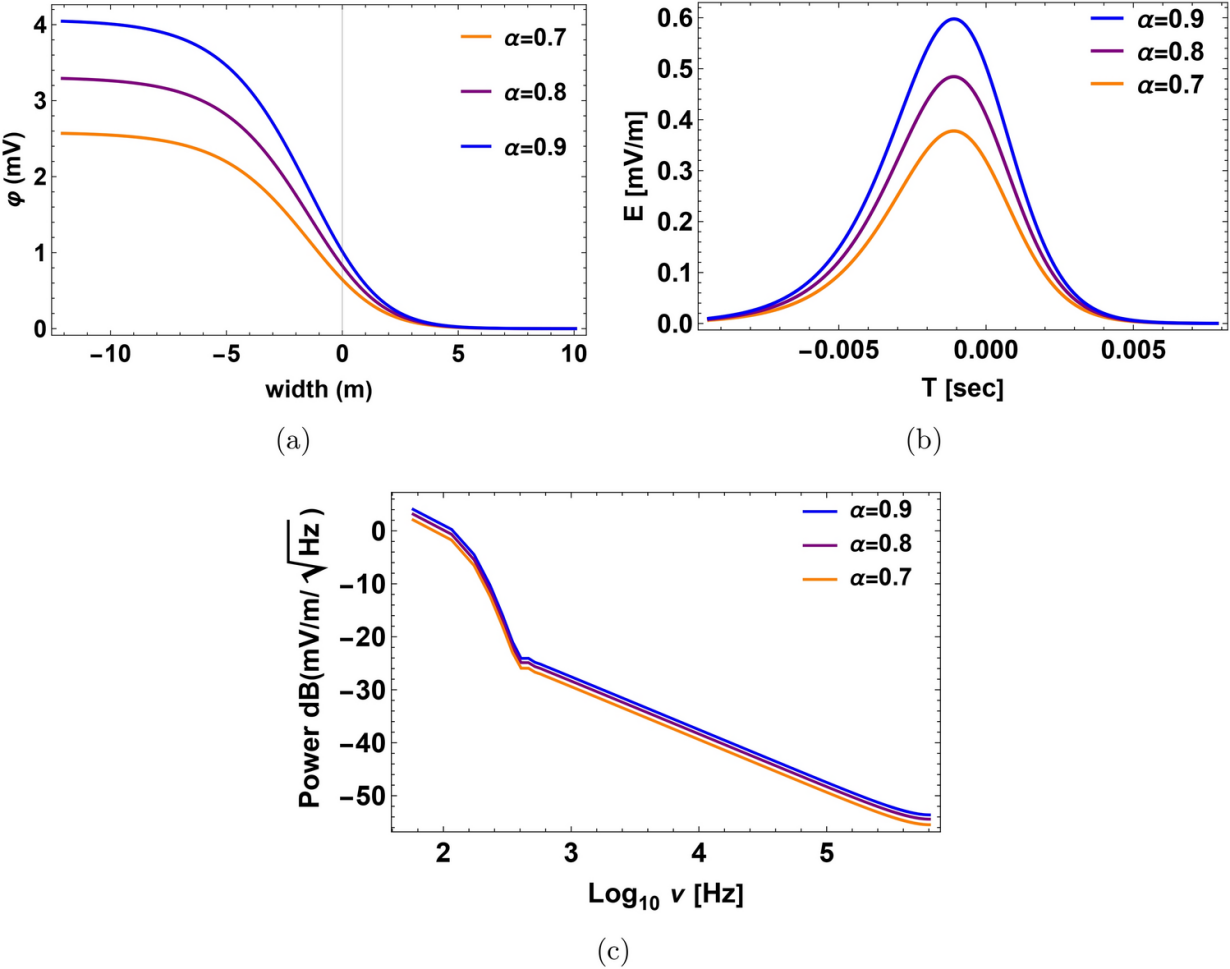
Profile of the Double layer (DL) with its corresponding bipolar electric pulse and the FFT power spectra at different relative densities $\alpha$ at $\sigma _h=0.2$, $\sigma _o=0.1$ and $\beta =0.01$. (**a**) The DL electrostatic potential is expressed in millivolts (mV) with the width of the pulse in meters (m). (**b**) Associated electric field pulse with time duration in seconds (s). (**c**) The electric field power spectra obtained from the corresponding fast Fourier transform (FFT). The x-axis represents the frequency $log_{10}{\nu }$ in Hz, while the y-axis represents the strength of the electric field $dB(mV/m/\sqrt{Hz})$.

In Fig. 4c, the potential of DL pulse does not depend on the oxygen temperature ratio $\sigma _o$, since, the fast mode phase velocity $\lambda _1$ does not change with $\sigma _o$ approximately (c.f. Fig. 2c). In Fig. 4d, the DL electric potential becomes low with $\beta$. Physically, for low $\beta$, i.e., higher trapped electrons temperature, it means that the system is far away from the Maxwellian state, and the more electrons are trapped in the phase space (c.f. Fig. 2d). It creates a high charge separation between the phase space hole and the surrounding plasma. Thus, the charged particles are accelerated due to high DL electric potential. For high $\beta$, it means that the difference between the number of trapped electrons and free electrons becomes low. Therefore, the acceleration of charged particles is not strong enough due to the low DL electric field. The DL profiles with a spatial width and the associated electric field are depicted in Fig. 5. When the hydrogen relative density rises, the profile of the DL pulse, associated electric field, and time duration increase as shown in Fig. 5a, b. The output of the FFT of this electric pulse is a broadband electrostatic noise in the range (100–800) Hz as shown in Fig. 5c.

#### Slow mode

**Fig. 6:**
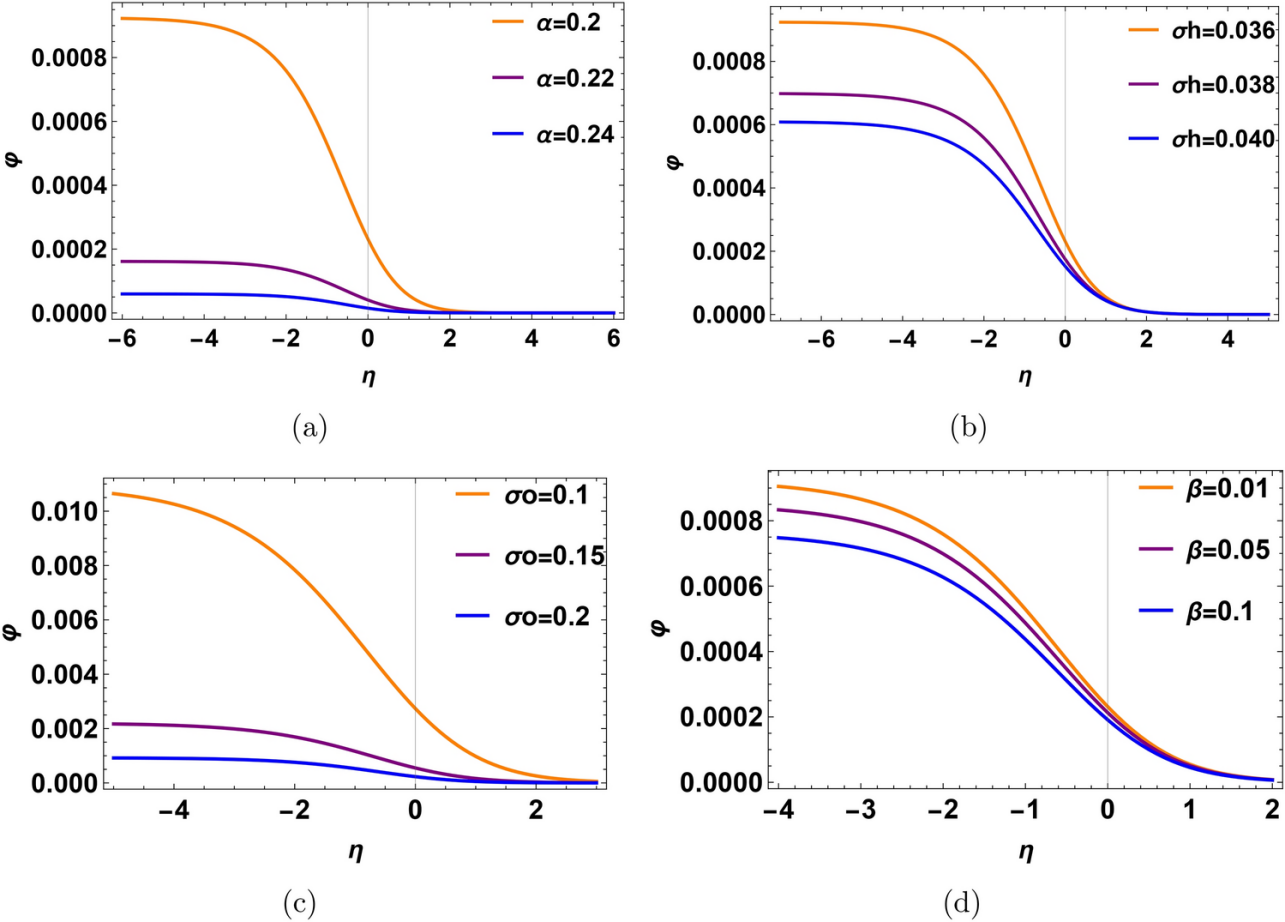
Profile of the electrostatic potential with different physical parameters in Double layer (DL). (**a**) The profile for the variation of density ratio $\alpha$ at $\sigma _h=0.036$, $\sigma _o=0.2$ and $\beta =0.01$. (**b**) The profile for the variation of temperature ratio $\sigma _h$ at $\alpha =0.2$, $\sigma _o=0.2$ and $\beta =0.01$. (**c**) The profile for the variation of temperature ratio $\sigma _o$ at $\alpha =0.2$, $\sigma _h=0.036$ and $\beta =0.01$. (**d**) The profile for the variation of temperature ratio of trapped coefficient $\beta$ at $\alpha =0.2$, $\sigma _o=0.2$ and $\sigma _h=0.036$.

Recalling that the slow mode corresponds to the oxygen ion time scale. In Fig. 6, we show the profile of DL electric potential with different plasma parameters. It is noticed that as the relative density $\alpha$ increases, the profile of DL potential decreases as shown in Fig. 6a. When the density ratio $\alpha$ increases, means that the phase velocity of oxygen ions $\lambda _2$ becomes low. So, the electric potential of the DL pulse is lower. Probably, when the hydrogen temperature ratio $\sigma _h$ rises, the DL profile becomes lower. Because, the rise in hydrogen thermal temperature enlarges the thermal energy gap between hydrogen and oxygen ions, reducing their effective interaction. Since our time scale is relative to oxygen ions, the DL profile decreases (c.f. Fig. 2b, 4b). Therefore, the amplitude of the DL pulse decreases, as shown in Fig. 6b. In Fig. 6c, the profile of DL rises when the oxygen temperature ratio $\sigma _o$ decreases because the phase velocity of oxygen ions $\lambda _2$ increases, so thermal energy increases, and the thermal separation between the ions temperature and electrons temperatures decreases. So, the potential difference and the amplitude of DL decrease. In Fig. 6d, for low $\beta$ (high trapped electron temperature), the system deviates significantly from a Maxwellian state; trapping more electrons leads to creating a strong charge separation. This results in a higher DL electric potential and stronger particle acceleration. Conversely, high $\beta$, means fewer trapped electrons relative to free electrons, leading to a weaker DL electric field and reduced particle acceleration (C.f. Fig. 4d).

**Fig. 7:**
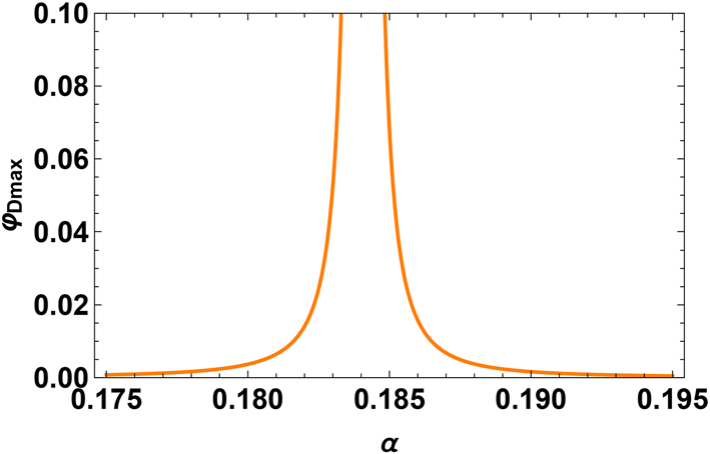
Profile of the double layer (DL) amplitude $\varphi _{Dmax}=\frac{4{C}^2}{25{A}^2}$ with relative densities $\alpha$ at $\sigma _h=0.036$, $\sigma _o=0.2$ and $\beta =0.05$.

In our investigation, the behavior of the system is much influenced by the parameter $\alpha$. The behavior of the field at the critical value of the density ratio-denoted as $\alpha _c$-showered a notable shift. This critical number represents a threshold wherein the dynamics of the system experience an essential change. Analytically determined from Eq. (21) in the text, the critical value of $\alpha _c$ results from zeroing the denominator of the double-layer amplitude formula. This mathematical condition finds the point at which the behavior of the system changes qualitatively. Furthermore, we quantitatively confirmed this critical value as shown in Fig. 7. These combined numerical and analytical methods guarantee the precision and dependability of $\alpha _c$ estimation. One important realization is that the nonlinear coefficient in the governing equation disappears at this particular value of $\alpha _c$. Thoroughly, consequences follow from this disappearance of the nonlinear coefficient: the amplitude of the wave solution diverges to infinity. This discrepancy emphasizes the physical relevance of $\alpha _c$ by matching with a singularity in the system. A fundamental feature of the model, the critical behavior at $\alpha _c$ indicates a point of unpredictability or transition. Accurate finding of this important point and capture of the related singularity in the system depend on the great accuracy in the approximation of $\alpha$. For example, $\alpha = 0.184067$. Such accuracy guarantees predictable and reliable numerical and analytical findings, thereby offering trustworthy knowledge of the important behaviors in the model. This thorough explanation clarifies the reason for the presentation of numerous significant digits for the value of $\alpha$ of this work.

21 $$\begin{aligned} \alpha _{c}=\frac{1-\frac{3 \lambda _2 ^2+3 \sigma {\textrm{o}}}{\left( \lambda _2 ^2-3 \sigma {\textrm{o}}\right) ^3}}{\frac{3 \rho {\textrm{h}}^3 \sigma {\textrm{h}}}{\left( \lambda _2 ^2-3 {\rho \textrm{h}} {\sigma {\textrm{h}}}\right) ^3}+\frac{3 \lambda _2 ^2 \rho {\textrm{h}}^2}{\left( \lambda _2 ^2-3 \rho {\textrm{h}} \sigma {\textrm{h}}\right) ^3}-1}. \end{aligned}$$

**Fig. 8:**
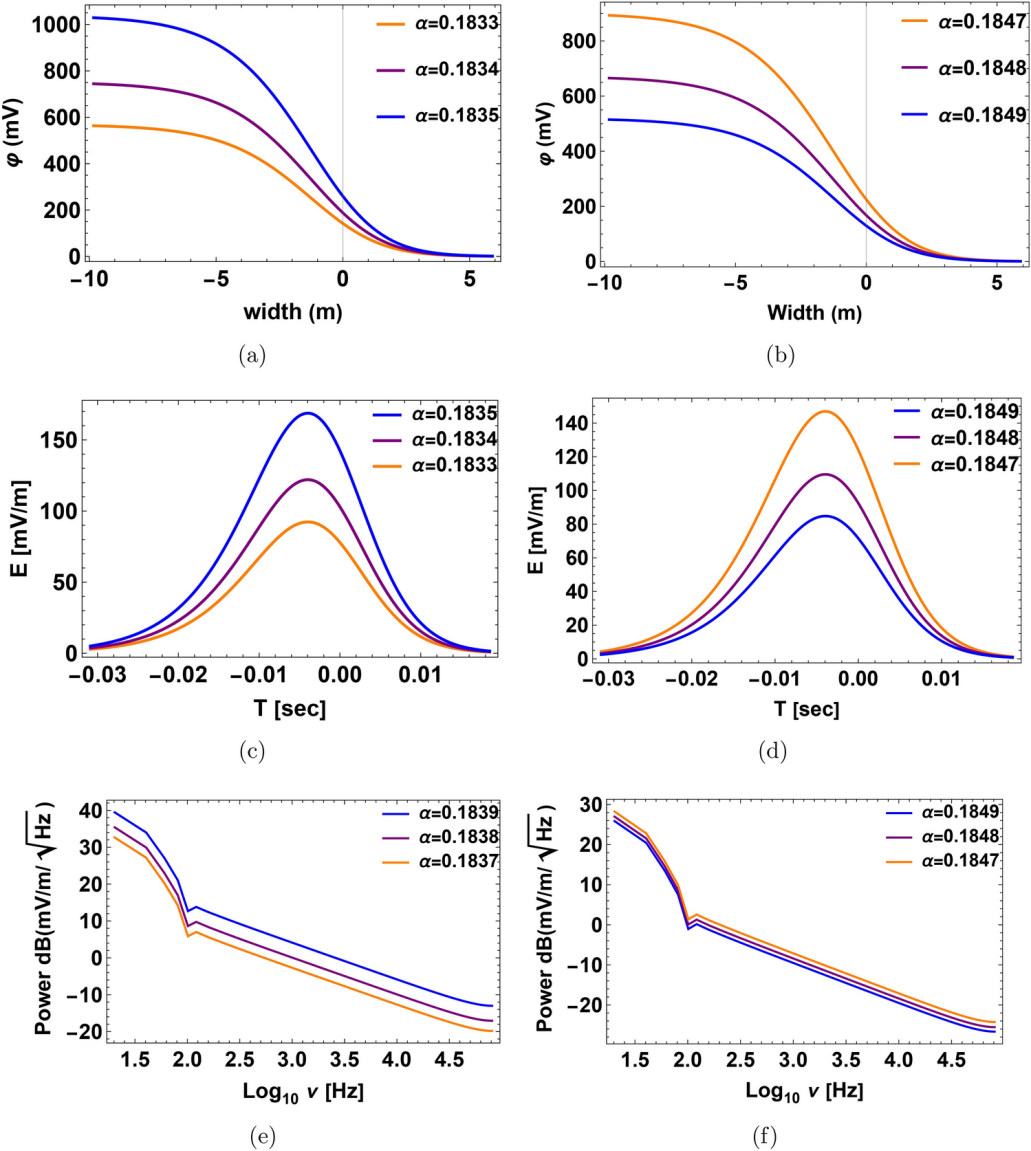
Profile of the double layer (DL) with its corresponding electrostatic potential, bipolar electric pulse and the FFT power spectra at different relative densities $\alpha$ at $\sigma _h=0.036$, $\sigma _o=0.2$ and $\beta =0.05$. (**a,b**) The DL electrostatic potential is expressed in millivolts (mV) with the width of the pulse in meters (m) before and after the critical relative density $\alpha _c$ respectively. (**c,d**) Associated electric field pulse with time duration in seconds (s) before and after the critical relative density, $\alpha _c$ respectively. (**e,f**) The electric field power spectra obtained from the corresponding fast Fourier transform (FFT). The x-axis represents the frequency $log_{10}{\nu }$ in Hz, while the y-axis represents the strength of the electric field $dB(mV/m/\sqrt{Hz})$ before and after the critical relative density, $\alpha _c$ respectively.

Significant behavior of the DL pulse and related electric field is found around the critical density ratio $\alpha _{c} \thickapprox$ 0.184067 in the slow mode that is found analytically by Eq. (21) numerically from Fig. 7. There is a clear amplification in both the electrostatic potential and the electric field as one approaches the critical point, producing a peak in amplitude as close to $\alpha _c$. It may be claimed that the non-linear coefficient *A* approaches zero. When the density ratio $\alpha$ approaches a critical value $\alpha _{c}$. The pulse profile, electric field strength, and time duration usually increase when the relative density $\alpha$ rises before this critical point $\alpha _{c}$, as shown in Fig. 8a, c, e respectively. When the relative density of hydrogen increases after the critical point $\alpha _c$, the profile of the DL pulse, associated electric field, and the power spectra decrease, as shown in Figs. 8b, d, f respectively.

**Fig. 9:**
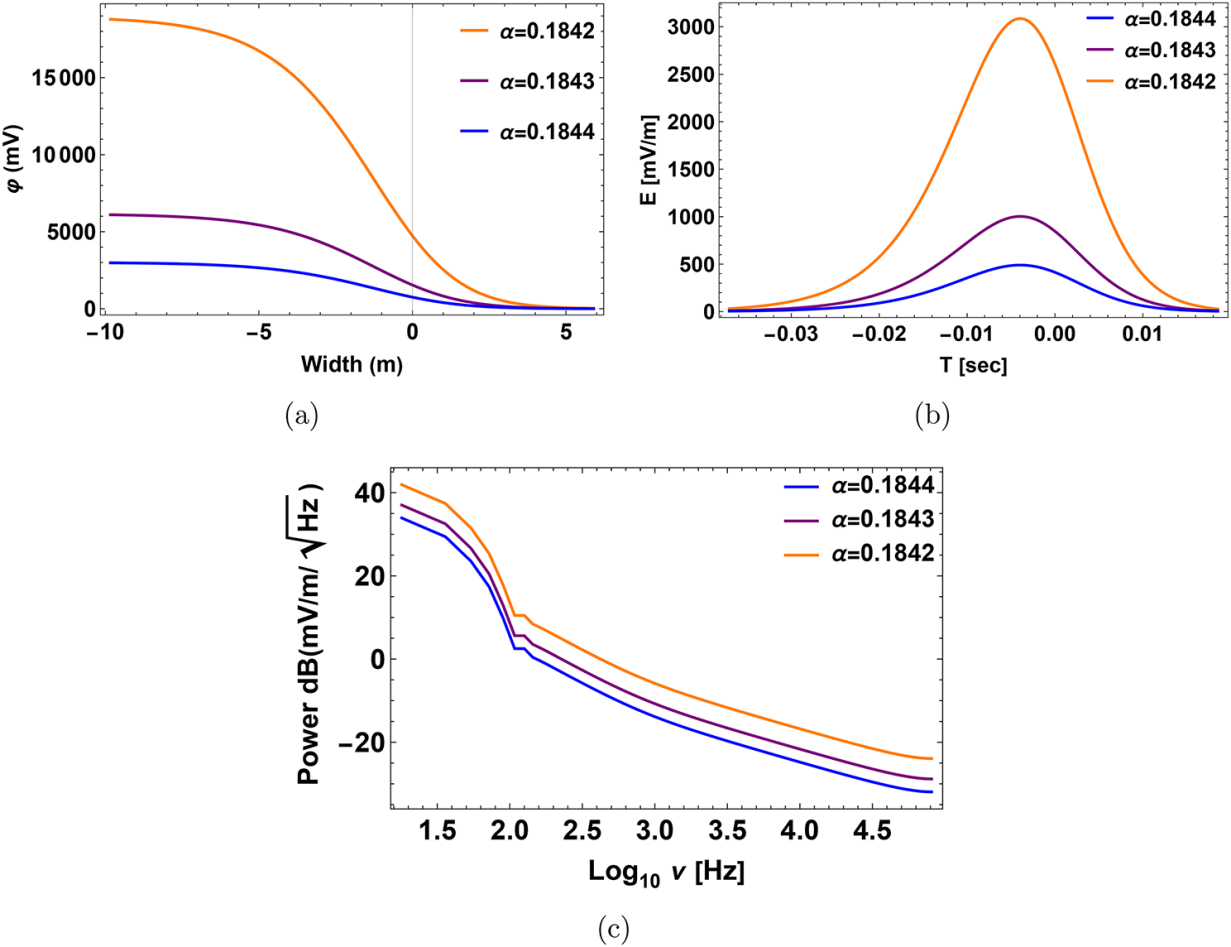
Profile of the double layer (DL) with its corresponding electrostatic potential, bipolar electric pulse, and the FFT power spectra at different relative densities $\alpha$ at $\sigma _h=0.036$, $\sigma _o=0.2$ and $\beta =0.05$. (**a**) The DL electrostatic potential is expressed in millivolts (mV) with the width of the pulse in meters (m). (**b**) Associated electric field pulse with time duration in seconds (s). (**c**) The electric field power spectra obtained from the corresponding fast Fourier transform (FFT). The x-axis represents the frequency $log_{10}{\nu }$ in Hz, while the y-axis represents the strength of the electric field $dB(mV/m/\sqrt{Hz})$.

There is a clear amplification in both the electrostatic potential and the electric field as one approaches the critical point, producing a peak in amplitude. Furthermore, the pulse’s temporal duration shows a small rise before maximum, corresponding with the peak in amplitude for a certain $\alpha$ as shown in Fig. 9a. As the associated graphs demonstrate, the FFT for this electric pulse, from 3000 mV/m to 20000 mV/m, produces a broad spectrum of electrostatic noise with frequency components ranging from 30 Hz to 100 Hz as shown in Fig. 9b, c. This trend draws attention to the nonlinear dynamics close to the critical density ratio. At this point, the system experiences strong electrostatic reactions before switching to a lower pulse profile. The previous statement implies that the nonlinear response decreases as the system gets closer to the density ratio threshold, indicating that it may transition to a nonlinear regime that is clear in the behavior of the electrostatic potential.

The use of Fast Fourier Transform (FFT) in the context of single-solitary structure, such as ion-acoustic solitary waves (IASW), is not ideal for analyzing their inherent properties. Actually, solitary waves are localized, non-periodic structures, and FFT is fundamentally designed to analyze signals that are periodic or have repeating patterns in time or space. Applying FFT to solitary structures can obscure key wave features because the method decomposes the signal into sinusoidal components, which may not effectively represent the characteristics of a solitary pulse. Indeed, the FFT will primarily provide information on the frequency content of the solitary waves, but it does not convey the full nonlinear characteristics of the wave, such as the amplitude, width, and localized nature of the pulse. By applying FFT to a solitary wave, one can extract the dominant frequency components that describe the solitary pulse. This information can be useful in identifying the characteristic frequency of the solitary structure’s propagation. Understanding the frequency components may help relate the solitary pulse to underlying plasma oscillations. We could validate our predictions by comparing the FFT results with the frequency observation of solitary waves. If a solitary wave produces a specific dominant frequency, this could be matched to theoretical expectations for specific ion-acoustic waves, thereby enriching our knowledge of wave dynamics in that environment.

## Summary and conclusion

A fluid model and perturbation technique are used to investigate phase space electron hole observation as a nonlinear electrostatic wave in the Venusian upper mantle boundary. We obtained two distinct modes, i.e., the slow and fast modes. Two analytical solutions representing the DL fluctuations and solitary pulses for each mode by different boundary conditions. We studied each mode in two analytical solutions (solitary and DL structures)^45^. It is estimated that plasma parameters such as relative density $\alpha$, hydrogen temperature ratio $\sigma _h$, oxygen temperature ratio $\sigma _o$, and trapped electron coefficient $\beta$ have an effect on the profile of the wave such as electrostatic potential, electric field, time scale, frequency, and power spectra in both DL fluctuations and solitary pluses. Here are the key findings from the paper:

1. As the density ratio $\alpha$ increases, the phase velocity $\lambda _1$ of the hydrogen ion, together with the electrostatic potential and the electric field in both solitary and double-layer (DL) structures, all increase in the fast mode. In slow mode, the phase velocity $\lambda _2$ of the oxygen ion, the electrostatic potential, and the electric field in the double-layer (DL) structure diminish till the critical point.
2. The phase velocity $\lambda _1$ of the hydrogen ion notices a considerable increase as the hydrogen temperature ratio $\sigma _h$ grows. On the other hand, the phase velocity $\lambda _2$ of the oxygen ion shows a slight increase, which is considered to be an insignificant increase. The electrostatic potential and electric field in double-layer (DL) structures and solitary structures both decrease in the fast mode and the slow mode, respectively.
3. As the oxygen temperature ratio $\sigma _o$ increases, the phase velocity $\lambda _1$ of the hydrogen ion constant and the phase velocity $\lambda _2$ of the oxygen ion increase. In fast mode, the electrostatic potential and electric field in both the solitary and double-layer (DL) structures have not changed. Conversely, the electrostatic potential and the electric field double-layer (DL) structure decrease in the slow mode.
4. As the trapped electron coefficient $\beta$ increases, the phase velocities $\lambda _1$ and $\lambda _2$ do not change. In the fast mode, the electrostatic potential and electric field in the solitary structure are almost increased. The electrostatic potential and the electric field in the double-layer (DL) structure decrease slightly.
5. We also found the critical value $\alpha _c$, in slow mode DL structures, where this fundamental value determines the amplification of the electrostatic potential inside the system. Relative density parameter influences the DL slow mode behavior, which greatly increases the electrostatic potential. Studying space plasma in environments like Venus requires a knowledge of this fundamental value especially. Understanding how energy is distributed and transported within the planetary atmosphere depends on knowing the maximum possible value under these plasma circumstances. By affecting particle dynamics and energy dissipation, the enhanced electrostatic potential provides insightful analysis of space plasma activity near Venus. This knowledge could support more general planetary science study and assist in forecasting atmospheric events.
6. Finally, the FFT of the bipolar pulse shows electrostatic activity around 1-1.5 kHz, with decreasing frequency associated with increasing relative density $\alpha$. Our findings align with Doppler-shifted frequency measurements by PVO at 5.4 kHz (central ± 15%), indicating electric field values of $E = 15-20$ mV/m, duration $T = 1-1.6$ ms, and frequency $f = 1-1.5$ kHz (^13,21^). Despite the absence of simultaneous electric and magnetic field measurements, these results support mantle waves as ion acoustic waves (IAWs). The PSP has also discovered solitary structures on Venus’ upper mantle (^21,26,27^). Comparative investigations reveal identical electric field amplitudes and frequency, but slow-mode ion acoustic waves are in disagreement with the observation..

## Data Availability

The data that support the findings of this study are available from the corresponding author upon reasonable request.
